# Revelation of an important weakness in polio elimination efforts in Nigeria: a descriptive cross-sectional study of nomadic dynamics in Sokoto and Taraba States, May 2013

**DOI:** 10.11604/pamj.supp.2021.40.1.32542

**Published:** 2021-12-24

**Authors:** Nuruddeen Aliyu, Musa Kalamullah Bawa, Saheed Gidado, Chima Ohuabunwo, Lisa Esapa, Wiedad Roodly Archer, Adamu Sule, Halimatu Ayanleke Bolatito, Aisha Mamman, Adebola Olayinka, Muhammad Shakir Balogun, Kabir Ibrahim Getso, Mahmood Muazu Dalhat, Ahmed Suleiman Haladu, Usman Lawan Shehu, Patrick Mboya Nguku, Aminu Shehu, Shehu Abdulganiyu, Ndadilnasiya Endie Waziri

**Affiliations:** 1 African Field Epidemiology Network, Abuja, Nigeria,; 2 African Field Epidemiology Network, Kampala, Uganda,; 3 US Centers for Disease Control and Prevention, Atlanta, Georgia, Unites States,; 4 Ahmadu Bello University, Zaria, Nigeria,; 5 Ministry of Health, Kano State, Nigeria

**Keywords:** Nomadic populations, enumeration, oral polio vaccine, underserved settlements, hard-to-reach settlements

## Abstract

**Introduction:**

Operational gaps in the Global Polio Eradication Initiative implementation had been partly responsible for inadequate population immunity and the continued transmission of wild poliovirus in Nigeria before the African Region was declared polio-free in 2020. Missed opportunities to provide services in nomadic populations due to frequent mobility, lack of inclusion in microplans and the remoteness of their settlements were the major challenges. During May 2013 we conducted immunization outreach to nomadic and other underserved communities in Rabah LGA, Sokoto state, and Ardo Kola LGA, Taraba state, in Nigeria to identify and vaccinate children missed during supplemental immunization activities while identifying missed acute flaccid paralysis cases.

**Methods:**

An enumeration checklist and data collection instruments on Android cell phones were used to capture socio-demographic data and GPS coordinates on nomadic settlements, households, number of children aged <5 years, children previously missed for vaccination and their locations. Local guides led trained enumerators to underserved communities for the enumeration and vaccination. Data were analyzed using Microsoft Excel 2007.

**Results:**

A total of 324 settlements were listed for the two states, and 111 (34.3%) of these were identified as missed when compared with micro-planning for the most recent SIA. In these settlements, 3,533 households and 9,385 children aged <5 years were listed. We administered oral poliovirus vaccine to all 1,946 missed children during the recent or any supplemental immunization activities. Of these, 527 (27.1%) had never been vaccinated. We found no missed acute flaccid paralysis cases.

**Conclusion:**

Nomadic populations continue to be underserved, especially for vaccination services. This results in pockets of populations with low herd immunity and increased risk for poliovirus transmission. Community leaders and nomadic settlements should be included in the micro-planning of all supplemental immunization activities to ensure all children receive vaccination services.

## Introduction

The wild poliovirus (WPV) transmission remains endemic in two countries (Afghanistan, and Pakistan) [1-4]. Before WPV transmission was halted in Nigeria in 2020, persistent WPV transmission presented a risk for WPV re-introduction into polio-free countries in the region and beyond [5]. In 2002-2005, 21 previously polio-free countries were affected by importations of the WPV type 1 from Nigeria, including Indonesia, Somalia, and Yemen [5]. Many other exportations of WPV originating in Nigeria occurred into other countries up to 2014. Continued endemic WPV transmission in Nigeria resulted from chronic low immunity in children from multiple underserved communities. The nomadic Fulani pastoralist populations are one of the most challenging underserved communities in Nigeria [6].

The movement of nomads and migrant populations has been identified as a reservoir for the sustained transmission of poliovirus [7-9]. Because of their frequent mobility, remoteness of their settlements, and inadequate microplanning to include them, children of nomadic groups might be missed completely for vaccination or be only partially, intermittently reached during supplemental immunization activities (SIA) with oral poliovirus vaccine (OPV). Low immunization coverage in this group has led to a large, susceptible migrant population [7,10] Therefore, ensuring access to immunization services for nomadic populations was critical to Nigeria´s efforts to eradicate polio.

Given the high mobility of nomads, systematic surveillance data on the health status of these populations have been difficult to obtain. Most surveillance information is based on specific, often small-scale studies - each one providing information on a limited portion of the overall population and only for a short time window [11]. Our study was conducted as a small immunization outreach program to nomadic communities in two local governments in Sokoto and Taraba States; our objectives were to obtain micro-census data, identify missed AFP cases, and administer OPV to previously missed children.

## Methods

### Study area and population

**Figure 1:**
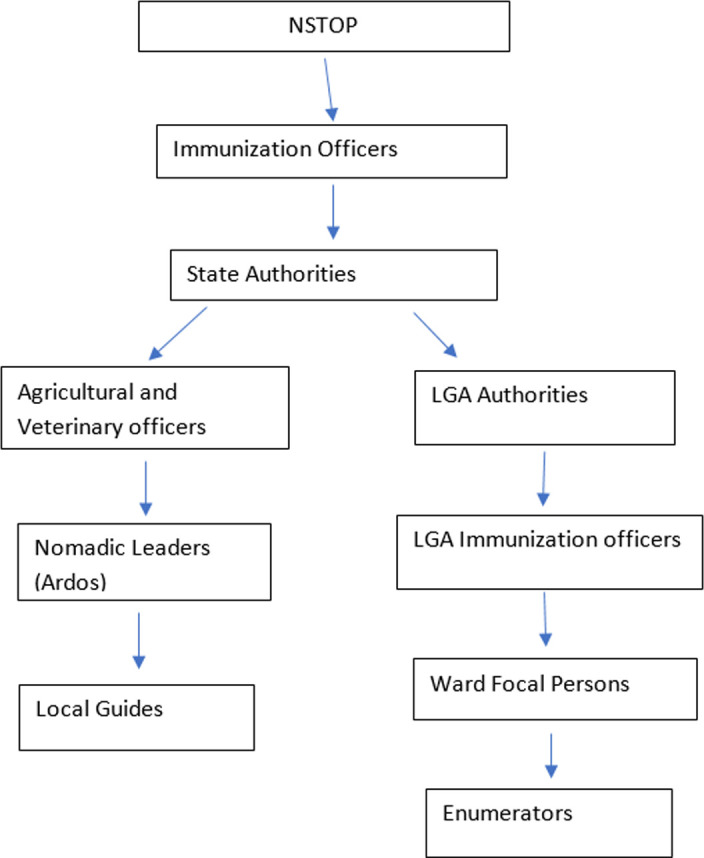
Flow chart showing stakeholders and the community engagement process

The study was conducted in Rabah Local Government Area (LGA) of Sokoto state and Ardo Kola LGA of Taraba state in northern Nigeria. These LGAs were classified as high-risk for polio transmission, given the presence of large numbers of underserved, nomadic, and hard-to-reach settlements. For advocacy, we visited the immunization authorities, traditional leaders and agricultural extension workers at the state level, and Ardos (nomadic leaders) at the state and LGA levels. The stakeholders were briefed on the planned enumeration of underserved communities (Figure 1). The study population was comprised of all children aged <5 years residing in households in the nomadic settlements in Rabah and Ardo Kola LGAs. We defined a settlement as a single, isolated household to a collection of households within a well-defined, small geographical boundary, with a recognized leader. Nomadic settlements were defined as settlements occupied by people who move from place to place in search of food and water for their animals and led by an Ardo. A missed settlement was defined as any settlement that was not visited by the SIA vaccination teams during the most recent SIA, or any settlement that had never been visited by the vaccination teams. We defined a household as a group of individuals who ate from the same cooking pot, and an OPV zero-dose child was defined as a child who had never received a dose of OPV [9] upon obtaining a recall history from a caretaker.

### Community engagement and settlement harmonization meeting

We held a planning meeting with the LGA immunization and primary health care team, local religious, traditional, nomadic, and village leaders from known remote settlements before starting enumeration and vaccination activities. Other participants included veterinary officials who are routinely involved in providing animal health services to these communities (Figure 1). The leaders from the nomadic and other underserved settlements provided a comprehensive line list of all settlements known to them and their population estimates in the vicinity of where they resided. As remote settlements are often not fully captured in LGA records, this list was added to or harmonized with the existing settlement list (micro-plan) used by the LGA to plan SIAs. The harmonized list included all known nomadic, scattered, and border settlements, with estimates of the number of children aged <5 years in each settlement. The harmonized settlement list was used for planning visits to all settlements in each LGA [9].

### Composition and training of field personnel

Field teams were comprised of data collectors who were veterinary and agricultural staff and officers; they served as vaccinators and local guides. Local leaders provided the names of field guides who assisted teams in locating the remote settlements in their areas. One field team was assigned to each of the 21 total wards in both LGAs where the fieldwork was conducted. The guides and data collectors received training on conducting enumeration of residents of each settlement, vaccine and cold chain management techniques, OPV vaccination procedures, using Android mobile telephones with global positioning system (GPS) capability, searching methods for additional settlements not included in the harmonized settlement line list, and conducting an active search for AFP cases in the households of the settlements.

### Study instruments and design

We conducted this descriptive cross-sectional study, using predesigned settlement enumeration tools and forms [9] on Android mobile phones to capture socio-demographic data on settlements, households, vaccination status of children aged <5 years, AFP cases in children aged <15 years, and their settlements´ GPS coordinates using Android GPS-enabled mobile phones.

### Data collection

Field guides led the data collectors to listed underserved settlements. Data were collected, using the enumeration checklist tool and GPS Android phones. Data collected for each settlement included GPS coordinates, socio-demographic data of households, the number of children aged <5 years in each household and their vaccination status, the number of children missed during recent SIA, and the number of newly identified AFP cases and their GPS coordinates.

The data collection teams used hired vehicles, motorcycles, and canoes as needed to reach hard-to-reach and remote locations. The enumeration teams aimed to visit a settlement one day after SIA vaccination teams were to have visited that particular settlement. LGA Vaccine and Cold Chain Officers provided the teams with OPV, vaccine carriers, and ice packs on a daily basis to administer doses where needed. These field activities were conducted during the May 2013 SIA and lasted for four days. The field teams visited the known and recently discovered settlements, using the harmonized settlement list; they enumerated children aged <5 years and assessed their recent SIA OPV vaccination status by checking their fifth finger on the left hand. Any child found to be unmarked was classified as “missed”, and the child was vaccinated with OPV by the teams.

### AFP case search

The field teams searched for AFP cases in every household they visited, using the standard World Health Organization case definition for AFP [12]. If a suspected AFP case was identified, patient demographic data, OPV vaccination status, and the date of paralysis onset were collected. Suspected AFP cases identified during the enumeration activity were then compared to the AFP line list of cases at the LGA. Any suspected case not included in the LGA´s line list was considered an unreported AFP case and was reported to the LGA Disease Surveillance and Notification Officer for investigation and follow-up.

### Daily evening review meeting

The field team held a review meeting with the LGA immunization and primary health care team officers every evening of the SIA and survey to discuss findings, issues, and challenges. Arrangements for vaccines and other logistics for the next day were also discussed.

### Data management

We used a data tool template designed for this assessment and Android mobile devices to collect the data. Any updated line lists of settlements were provided to LGA officers to update future SIA micro-plans with the inclusion of recently identified settlements and groups. Uploaded GPS data were used for mapping nomadic/underserved settlements and AFP cases. Data were analyzed using Microsoft Excel 2007.

### Ethical considerations

The National Polio Emergency Operations Centre approved this assessment as a non-research programmatic activity and CDC project determination was obtained. All field activities were conducted with the consent of the LGA Primary Health Care, the community and settlement leaders. The children`s parents or caregivers provided verbal permission to vaccinate their children.

## Results

Field visits were conducted in all wards of Rabah (11) and Ardo Kola LGAs (10). In Rabah LGA, 77 nomadic or underserved settlements were enumerated, of which seven (9.1%) were missed in recent SIA using the previous micro-plan (Table 1).

**Table 1:** population characteristics of oral polio vaccine eligible children in underserved and nomadic communities of Rabah LGA, Sokoto State, Nigeria

Ward name	Number of underserved settlements visited and enumerated	Number of underserved households visited and enumerated	Number of children under 5-years of age enumerated	Children who have never been vaccinated (zero-doses) n (%)
Gandi 1	6	70	157	15 (9.6)
Gandi 2	10	82	351	34 (9.7)
Goddodi	8	90	257	3 (1.9)
Kurya	6	180	411	19 (2.2)
Maikujera	6	46	129	5 (3.9)
Rabah	10	269	669	8 (1.2)
Rara	6	157	427	0 (0)
Tofa	6	102	156	8 (5.1)
Tsamiya	5	53	106	9 (8.5)
Tursa	6	100	264	3 (1.1)
Yartsakuwa	8	147	276	11(4.6)
Total	77	1,296	3,203	115 (3.6)

**Figure 2:**
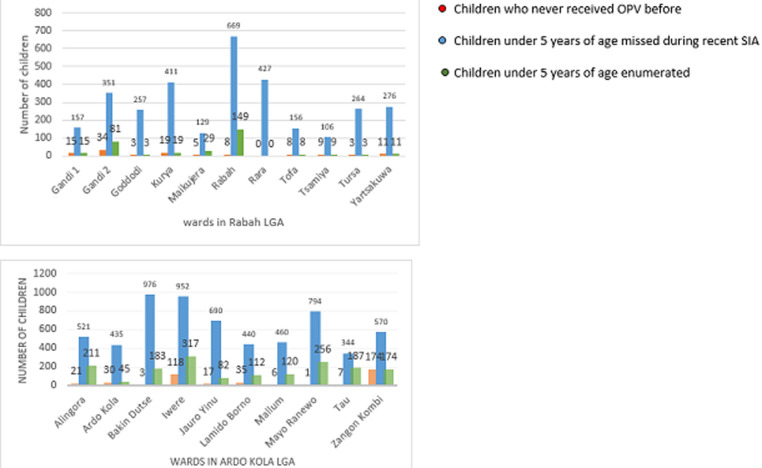
The number of children aged <5 years enumerated, missed, and those who have never been vaccinated before in Rabah and Ardo Kola LGAs, 2013

The average number of underserved settlements was 7 (range 5-10). A total of 1,296 households with 3,203 children aged <5 years were enumerated. Based on parental recall and no evidence of finger marking for the SIA, 259 (8.1%) of the children counted were missed during the recent SIA, and 115 (3.6%) were never vaccinated through routine immunization or OPV SIAs (Table 2). The Rabah ward had the highest number of nomadic or underserved households (269, 20.8%) and children aged <5 years (669, 20.9%), whereas the Maikujera ward had the lowest number of nomadic or underserved households (46, 4%). The Gandi 2 ward had the highest number of zero-dose children (34, 9.7%). In Ardo Kola LGA, 247 nomadic or underserved settlements were enumerated, of which 104 (42.1%) were missed by the recent SIA using the previous micro-plan. The average number of underserved settlements was 25 (range 19-33). A total of 2,237 households with 6,182 children aged <5 years were enumerated. Of those, 1,687 (27.3%) children were missed during the recent SIA, and 412 (6.7%) had never been vaccinated before (Figure 2). Tau ward had the highest proportion of children missed during the recent SIA (187; 54%). Zangon Kombi ward had the highest number of children who had never been vaccinated (174; 31%) (Table 3). All the children missed by the recent or any SIA were vaccinated with OPV. However, no unreported AFP cases were identified in either LGA during this four-day operation.

**Table 2:** number of missed settlements, households, and children aged <5 years identified by the enumeration teams that were not included in the micro-plan in Rabah LGA, Sokoto state by ward, Nigeria, 2013

Ward	Name of settlement identified by the enumeration team that were not included in SIA micro-plan	Number of underserved households visited in settlements	Number of missed children aged <5 years identified and vaccinated by the enumeration team
Gandi 2	Gallawa	4	11
	Yan Marake	8	28
	Rafin Namaru	11	42
Maikujera	Tulluwargero	10	19
	Dandutsi	6	10
Rabah	Gidan Amale	30	69
	Gidan Bugaje	20	80
Total	7	89	259

Note: All missed children were vaccinated by the enumeration teams

**Table 3:** population characteristics of oral poliovirus vaccine-eligible children in underserved and nomadic communities by ward, Ardo Kola LGA, Taraba State, Nigeria, 2013

Wards	Number of underserved settlements visited and enumerated	Number of underserved households visited and enumerated	Number of children under 5-years of age enumerated	Children under 5-years of age found to be missed during the recent SIA n (%)	Children who have never been vaccinated before (zero-doses) n (%)
Alingora	23	155	521	211 (40.5)	21 (4.0)
Ardo Kola	28	134	435	45 (10.34)	30 (6.9)
Bakin Dutse	33	368	976	183 (18.75)	3 (0.3)
Iwere	26	281	952	317 (33.3)	118 (12.4)
Jauro Yinu	19	259	690	82 (12.6)	17 (2.5)
Lamido Borno	26	186	440	112 (25.45)	35 (8.0)
Mallum	25	175	460	120 (26.1)	6 (1.3)
Mayo Ranewo	21	255	794	256 (32.2)	1 (0.1)
Tau	19	136	344	187 (54.4)	7 (2.0)
Zangon Kombi	27	288	570	174 (30.5)	174 (30.5)
Total	247	2,237	6,182	1,687 (27.3)	412 (6.7)

Note: All missed children were vaccinated by the enumeration teams

## Discussion

The lack of health and social services for these nomadic and underserved communities is evidenced by the identification of a large number of new settlements that had never been visited for vaccination, large numbers of children missed during recent SIA, and large numbers of children who had never received OPV before by any means. However, the study did not attempt to estimate the length of time for which the settlements were missed. Omar [13] has argued that underserved and nomadic populations that are difficult to access need particular attention for health services delivery, as they are often excluded from routine health services available in accessible areas. Wards in the two sampled LGAs varied in the number of underserved settlements enumerated during the survey, with Ardo Kola having a much larger average and upper limit than Rabah LGA, indicating LGA differences in the quality of the micro-plans. Bakin Dutse of Ardo Kola LGA had the highest number of underserved settlements identified, while Tsamiya ward of Rabah LGA had the lowest number.

A large number of these underserved settlements that were identified were missed in the recent SIA, further indicative of gaps in micro-planning. Key factors responsible for these disparities included inadequate micro-planning, limited resources for logistic support, and the lack of community leader engagement in the micro-planning process [11]. The Iwere ward of Ardo Kola LGA had the highest number of children missed, whereas the Maikujera ward of Rabah LGA had the lowest number of children missed. Iwere ward is one with the furthest distance away from the Ardo Kola capital. Children who are chronically missed during SIAs may jeopardise global polio eradication and threaten efforts in controlling and eliminating other vaccine-preventable diseases [14-17].

Our study in the two LGAs showed that children living in underserved areas and nomadic populations might be missed completely or intermittently vaccinated during polio SIAs due to their frequent mobility and remoteness of their settlements [13]. The nomadic routes have been associated with long-term transmission of poliovirus in northern Nigeria, including the north-central states [18,19]. It should be noted that the missed children were not as a result of parental refusal: all missed children were vaccinated consequently during our study with parental consent, which illustrates that parents in those areas are willing to accept vaccination services for their children when reached.

Overall, 412 (24%) children vaccinated in the scattered, nomadic, and other underserved settlements had never been vaccinated before. Even though a review of primary health care services was not part of our assessment, children residing in the remote nomadic, scattered, and border settlements were likely lacking ready access to routine immunization (RI) services, including outreach. We referred children younger than 12 months in the zero-dose group for RI services and sensitized their parents and caregivers on the importance of vaccination overall and seeking RI services that are typically concentrated in urban settlements. Additionally, constant mobility of nomadic communities further restricts their access to RI and essential health services, and could make efforts to reach them with outreach RI services difficult [20]. Nomads also have much less access to safe drinking water and formal education [10,21], therefore identifying these settlements could improve access to other services. The training and engagement of multidisciplinary and culturally acceptable local personnel who can reach underserved communities provides an available workforce that could be mobilized promptly to respond to various health issues in these communities.

Despite conducting active case searches for AFP, we did not find any missed current AFP cases; in a more extensive endeavour, Gidado et al. found clusters of unreported suspect AFP cases in some settlements [8]. Other studies have also revealed difficulties in integrating nomads into systems of disease surveillance [14,22,23].

Our findings have limitations. Firstly, our enumeration may have missed some settlements and children because of the remoteness and scattered nature of their households; however, every effort was made to ensure that no child is missed by consulting appropriate community leaders and the search for settlements based on field interviews of settlement leaders was extensive. In the future, a similar exercise could be strengthened by satellite imagery and mapping of enumerated households. Secondly, we were unable to collect socio-economic information of the households and children aged <5 years in the underserved populations. Thus, we could not establish a relationship between lack of vaccination in recent SIA and socio-economic status.

## Conclusion

By engaging community leaders, a large number of previously unidentified settlements were found with children who had missed polio vaccination. Unvaccinated nomadic children may have contributed to the maintenance and transmission of poliovirus in Nigeria. Sustaining access to this underserved population via SIA and RI activities will increase overall population immunity and prevent poliovirus transmission. Governments should intensify their efforts to involve traditional leaders and non-public health actors such as veterinarians and agriculture officers when planning health services for nomads and other underserved populations.

### What is known about this topic

- Nomadic and hard-to-reach communities are usually underserved in terms of health and social services;
- Nomadic children are completely missed or intermittently covered during polio Supplementary Immunization Activities (SIA) due to their frequent mobility and remoteness of their settlements;
- Most underserved communities are readily receptive to health services, including vaccination.

### What this study adds

- We found a large number of new settlements and households with many children aged < 5 years who have never been reached previously with vaccination services (i.e., caretaker recall history of zero poliovirus vaccine doses); all of them were vaccinated during the visits and added to the micro-plans for subsequent polio SIAs;
- Differences have been noted between wards within the same LGA but also between the two study LGAs in terms of children missed during the recent SIA. The most difficult-to-access wards had the highest number of children missed during the recent SIA and those who have never been vaccinated.
